# Asperosaponin VI promotes bone marrow stromal cell osteogenic differentiation through the PI3K/AKT signaling pathway in an osteoporosis model

**DOI:** 10.1038/srep35233

**Published:** 2016-10-19

**Authors:** Ke Ke, Qi Li, Xiaofeng Yang, Zhijian Xie, Yu Wang, Jue Shi, Linfeng Chi, Weijian Xu, Lingling Hu, Huali Shi

**Affiliations:** 1 Hangzhou Medical College Binwen Road,Hangzhou,310053, China.; 2 Stomatology Hospital, School of Medicine, Zhejiang university, Yan’an Road, Hangzhou 310006, China

## Abstract

Asperosaponin VI (ASA VI), a natural compound isolated from the well-known traditional Chinese herb *Radix Dipsaci*, has an important role in promoting osteoblast formation. However, its effects on osteoblasts in the context of osteoporosis is unknown. This study aimed to investigate the effects and mechanism of ASA VI action on the proliferation and osteogenic differentiation of bone marrow stromal cells isolated from the ovariectomized rats (OVX rBMSCs). The toxicity of ASA VI and its effects on the proliferation of OVX rBMSCs were measured using a CCK-8 assay. Various osteogenic differentiation markers were also analyzed, such as ALP activity, calcified nodule formation, and the expression of osteogenic genes, i.e., ALP, OCN, COL 1 and RUNX2. The results indicated that ASA VI promoted the proliferation of OVX rBMSCs and enhanced ALP activity and calcified nodule formation. In addition, while ASA VI enhanced the expression of ALP, OCN, Col 1 and RUNX2, treatment with LY294002 reduced all of these osteogenic effects and reduced the p-AKT levels induced by ASA VI. These results suggest that ASA VI promotes the osteogenic differentiation of OVX rBMSCs by acting on the phosphatidylinositol—3 kinase/AKT signaling pathway.

Osteoporosis (OP), characterized by low bone mass and compromised bone strength, is a skeletal disease that commonly affects older, postmenopausal or estrogen-deficient women. The disease is characterized by a high rate of bone remodeling, with a higher rate of bone resorption than bone formation^1^. OP increases the risk of fractures, loss of independence, and mortality, and it can lead to high health care costs^2^,^3^,^4^. OP management focuses on maintaining the balance between bone formation and bone resorption. Bone marrow stromal cells (BMSCs) give rise to osteoblasts that can form new bone tissues^5^,^6^. In the aging population, osteogenic differentiation of BMSCs decreases, leading to a significant reduction in bone formation^7^. Therefore, promoting the proliferation and osteogenic differentiation of BMSCs is crucial for developing OP treatment strategies. The current treatments for this condition are predominantly drug-based therapies, e.g., calcitonin, bisphosphonates, selective estrogen receptor modulators, and the human monoclonal antibody denosumab^8^. However, the use of these agents has been associated with an increased risk of osteonecrosis of the jaw^9^, fatal stroke^10^, increased bone turnover rather than osteoclast suppression^11^, and cancer^12^. Therefore, it is necessary to identify safer and more effective anti-OP agents.

Natural compounds derived from plants are resources for potential anti-OP drugs^13^,^14^,^15^. *Radix Dipsaci*, the dried root of *Dipsacus asper Wall*, is a kidney-tonifying herbal product with a long history of safe use for the treatment of bone fractures and joint diseases in China^16^. *Radix Dipsaci* extract prevented bone loss in rats induced by simulated microgravity^16^ and ovary excision^17^. In addition, *Radix Dipsaci* total saponins induced osteoblastic differentiation through a bone morphogenic protein (BMP)-2/mitogen-activated protein kinase (MAPK)/SMAD1/5/8-dependent runt-related transcription factor 2 (RUNX2) signaling pathway^18^.

Asperosaponin VI (ASA VI), a triterpenoid saponin, is considered to be the main bioactive component of *Radix Dipsaci*. Previous studies have used it as a quality control standard for *Radix Dipsaci*^19^. Currently, there are only a few reports on the osteogenic effects of ASA VI and only one report of it enhancing the proliferation and differentiation of MC3T3-E1 cells and primary rat osteoblasts^20^. There have been no reports to date on the osteogenic effects of ASA VI on BMSCs. The phosphatidylinositol—3 kinase (PI3K)/AKT signaling pathway is known to play a regulatory role in the survival, proliferation, migration, and differentiation of BMSCs^21^, and both *in vivo* and *in vitro* studies have indicated that the PI3K/AKT signaling pathway is involved in the inhibition of OP^22^. Furthermore, ASA VI has been reported to protect cardiac myocytes from hypoxia-induced apoptosis through the activation of the PI3K/AKT pathway^23^. In this study, we found that ASA VI played a role in the osteogenesis of BMSCs obtained from ovariectomized rats (OVX rBMSCs) and that this effect was dependent on the PI3K/AKT signaling pathway.

## Materials and Methods

### Reagents

ASA VI (purity >99%, National Institute for the Control of Pharmaceuticals and Biological Products, Beijing, China) was dissolved in phosphate-buffered saline (PBS) and stored at −20 °C. Dulbecco’s Modified Eagle’s Medium (DMEM) and fetal bovine serum (FBS) were purchased from Sigma (Sigma-Aldrich Co., USA). A CCK-8 kit was obtained from Dojindo (Kumamoto, Japan). Bicinchoninic acid (BCA) protein assay kit was obtained from Jiancheng (Nanjing, China)and alkaline phosphatase (ALP) activity assay kit was obtained from Wako (Japan). LY294002 was obtained from the Biyuntian Bioengineering Institute (Shanghai, China). Ascorbic acid phosphate, β-glycerophosphate, dexamethasone and alizarin red S were also purchased from Sigma (Sigma-Aldrich Co, USA).

### Animals and OVX models

Three-month-old, female Sprague-Dawley rats weighing between 240 and 260 grams were obtained from the Animal Experiment Center of Zhejiang University (Hangzhou, China). All animal experiments were performed in accordance with the Animal Care and Use Committee guidelines of Zhejiang province. The experiments of this study were approved by the Institutional Animal Care and Use Committee of Zhejiang University, Hangzhou, China. 40 rats were divided into two groups randomly (20 OVX group, 20 Sham group). After receiving an intraperitoneal injection of 300 mg of chloral hydrate per kg of body weight, the rats underwent ovariectomy. The sham operation were used as controls. All rats were then housed in cages at room temperature (23 °C) with controlled humidity (55%) and a 12-hour light/dark cycle^24^. After 12 weeks, the femur of 10 rats (5 per group) were dissected and scanned using micro-computer tomography (Bruken, Belgium) to acpuire three-dimensional imagery of their cancellous bone micro structure and for analysis. Sample parameter included Bone mineral density (BMD), Bone volume/Total volume (BV/TV), The trabecular spacing (Tb. Sp).

### Cell culture

30 rats (15 per group) were sacrificed by an overdose of chloral hydrate to obtain rBMSCs for culture. These rBMSCs cultures were prepared according to the protocol developed in the Caplan laboratory^25^. In brief, the tibia and femur were excised, and all attached tissues were carefully removed under sterile conditions. The bone marrow was extracted with an injection of DMEM containing 10% FBS, 100 U/ml penicillin, and 100 μg/ml streptomycin. Cells were plated on plates 60 mm in diameter and incubated at 37 °C in a humidified atmosphere with 5% CO_2_ for 24 hours before the first medium change. At 85% confluence, the cells were trypsinized (0.25% trypsin-EDTA, Sigma) and passaged into flasks at a ratio of 1:3. rBMSCs from the third passage were used in all experiments. For all subsequent experiments except the cell proliferation assay, the culture medium was replaced with an osteogenic medium supplemented with 10 mM β-glycerophosphate, 50 μg/ml ascorbic acid and 10^–8 ^M dexamethasone^26^.

### Detection of osteogenic capability

The osteogenic capability of OVX rBMSCs and SH rBMSCs was tested. After osteogenic induction, expression of alkaline phosphatase (ALP) were examined on day 7 and day 14 separately. The formation of calciumnodes was analyzed by alizarin red staining on day 21. The procedures were the same as that for the assay described following.

### Cell proliferation assay

OVX rBMSCs were seeded at a density of 2 × 10^3^ cells/well in 96-well plates (Corning, Shanghai, China) containing DMEM and ASA VI at various concentrations (0, 10^−4^, 10^−5^, 10^−6^, 10^−7^, and 10^−8 ^M), with 6 replicate wells for each concentration. After 24, 96, or 168 hours (1, 4, or 7 days), CCK-8 solution (10 μl/well) was added. The cells were then incubated in darkness for 1 hour at 37 °C. After the plates were shaken for 10 seconds, the absorbance at 450 nm was measured.

### ALP activity assay

OVX rBMSCs were seeded in 24-well plates (5 × 10^3^ cells/well) together with various concentrations of ASA VI (0, 10^−5^, 10^−6^, 10^−7^, and 10^−8 ^M), with 3 replicate wells for each concentration. After 5 or 10 days of osteogenic induction, the cells were lysed with 0.2% Triton X-100 on ice and then centrifuged at 14,000 rpm at 4 °C for 15 minutes. The supernatant was collected to measure ALP activity using an ALP activity kit, and protein concentrations were measured using a BCA protein assay kit^26^.

### Alizarin red staining and mineraNlization assay

To investigate the mineralization of the OVX rBMSCs, cells were seeded in 24-well plates (5 × 10^3^ cell/well) in osteogenic medium with LY294002 (50 μM) and/or ASA VI (10^−5 ^M), with 3 replicate wells per concentration. The cultures were maintained for 21 days, and the medium was changed every 3 days. The cells were washed twice with PBS and fixed in 98% ethanol for 20 minutes. Then, the cells were washed with PBS again, and 200 μl of alizarin red solution was added to each well and incubated for 15 minutes at 37 °C. After the nodules were imaged, 10% cetylpyridinium chloride was used to dissolve the nodules, and absorbance at 562 nm was measured^27^.

### Real-time PCR assay

Real-time PCR was performed to detect the expression levels of several significant osteogenic differentiation-related marker genes (i.e., ALP, osteocalcin [OCN], type 1 collagen [COL 1], and runt-related transcription factor 2 [RUNX2]). OVX rBMSCs were seeded in 6-well plates (4 × 10^4^ cells/well) in osteogenic medium with ASA VI (10^−5 ^M) and/or LY294002 (50 μM), with 3 replicate wells per concentration. After 48 hours of osteogenic induction, total RNA was extracted with Trizol reagent (Invitrogen Life Technologies, USA) in accordance with the manufacturer’s instructions. DNase I (Invitrogen, USA) was added to the samples to eliminate any DNA contamination. The RNA samples were then used to synthesize cDNA with a RevertAid First Strand cDNA Synthesis Kit (Thermo, USA) for ALP, OCN, COL 1, and RUNX2, and the data were normalized to GAPDH.

### Western blot analysis

The obtained OVX rBMSCs were cultured for 2 days in osteogenic induction conditions, as previously described. Then, they were washed 3 times with PBS and lysed with RIPA buffer (Wuhan Guge, China) on ice before being centrifuged at 12,000 g for 5 minutes. The supernatant was then collected, and protein concentrations were determined using a BCA protein assay kit; 50 μg of protein for each sample was boiled for 5 minutes and was then separated by SDS-PAGE and transferred onto a PVDF membrane (Millipore, Germany). The membranes were blocked with 5% BSA in TBST buffer and were subsequently incubated with primary antibodies against p-AKT overnight at 4 °C. Then, the membranes were washed 3 times in TBST for 15 minutes and incubated with secondary antibodies (1:3,000) for 30 minutes at room temperature. After visualization using enhanced chemiluminescence, the experimental band absorbance values were determined relative to the absorbance of β-actin.

### Statistical Analysis

All experiments were repeated at least in triplicate, and one-way ANOVA was used to compare differences among groups. *P* values < 0.05 were considered statistically significant.

## Results

### Micro-CT evaluation

**Figure 1:**
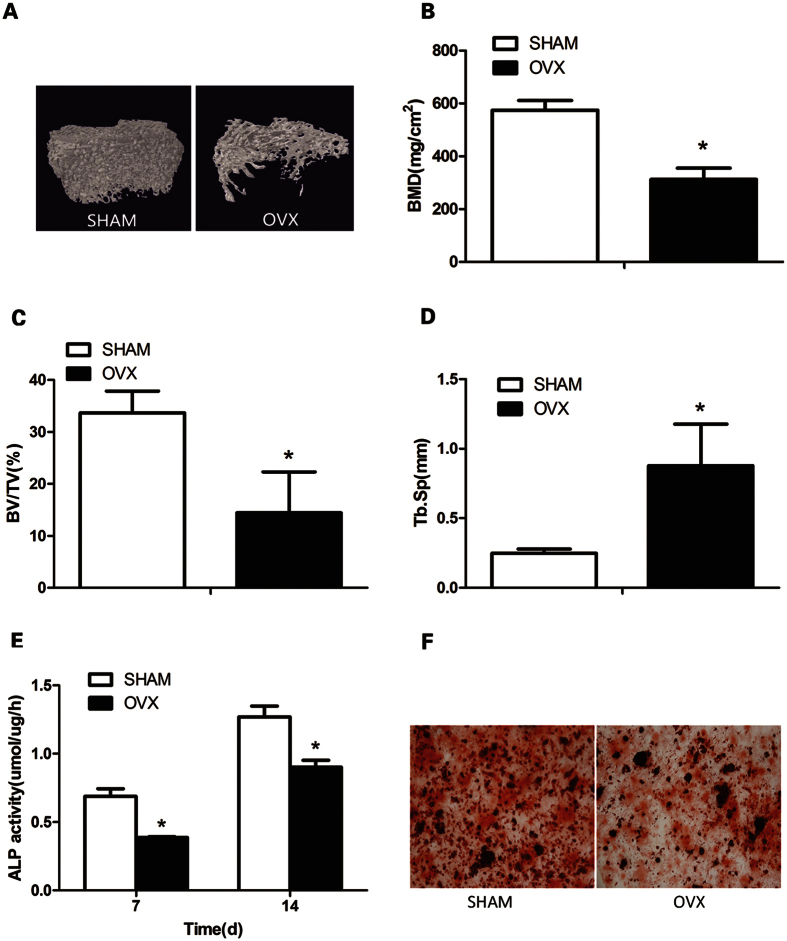
3D reconstructions of femoral metaphyseal trabecular structure were observed by micro-CT. (**A**) BMD (bone mineral density) of SHAM group and OVX group was determined by micro-CT. (**B**) BV/TV (Percent bone volume) of SHAM group and OVX group was determined by micro-CT. (**C**)Trabecular Sepa ration/Spacing of SHAM group and OVX group was determined by micro-CT. (**D**) The ALP expression of the two groups of BMSCs were observed on 7d and 14d. The OVX group was significantly lower than that of the SHAM group. (**E**) The calcium deposit formation of the two groups were determined by Alizarin red staining. The OVX group was significantly lower than that of the SHAM group. (**F**) The data are represented as the mean ± SD. *P < 0.5 vs the SHAM group.

Micro-CT three-dimensional imagery showed that in OVX group the bone microstructure of femur were damaged badly with the bone trabecula being sparser when compared to the Sham group (Fig. 1A). Meanwhile, OVX group showed a significant decrease in the BMD (Fig. 1B) and BV/TV (Fig. 1C), but Tb. Sp (Fig. 1D) was significantly higher (*P* < 0.05).

### Detection of osteogenic capability

The results showed that the OVX group was significantly lower than that of the Sham group, including the expression of ALP (*P* < 0.05)(Fig. 1E) and calcium deposit formation (Fig. 1F) (P < 0.05).

### Cell proliferation

**Figure 2:**
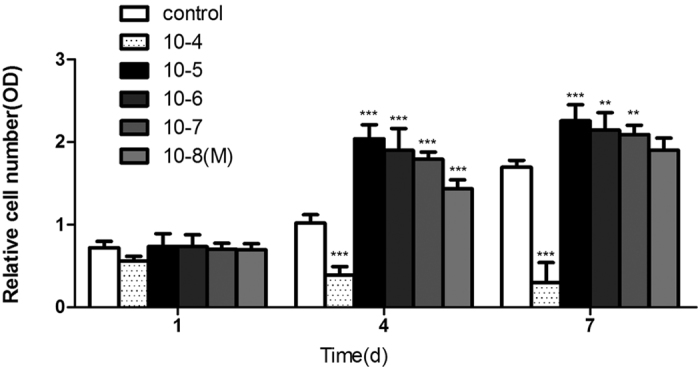
The effect of ASA VI on OVX rBMSC proliferation was analyzed at 1, 4, and 7 days, respectively. Cell proliferation was evaluated by a CCK-8 assay. The data are represented as the mean ± SD. **P < 0.01 vs the controls. ***P < 0.001 vs the controls.

OVX rBMSC proliferation was analyzed using a CCK-8 assay on days 1, 4, and 7. ASA VI did not affect cell proliferation on day 1. On day 4 and 7, ASA VI (10^−5^, 10^−6^, 10^−7^, and 10^−8 ^M) promoted the proliferation of OVX BMSCs in a dose-dependent manner, while ASA VI 10^−4^M inhibited cell proliferation (Fig. 2).

### ALP activity

**Figure 3:**
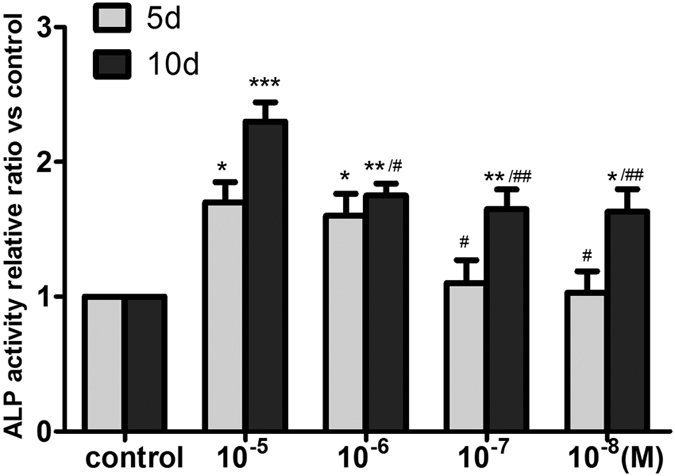
ALP activity was assessed using a commercial ALP kit after cells were cultured with various concentrations of ASA VI for 5 or 10 days. The data are represented as the mean ± SD. *P < 0.05, **P < 0.01, and ***P < 0.001 vs the controls; ^#^*P* < 0.05 and ^##^*P* < 0.01 vs ASA VI 10^−5 ^M.

ALP activity is an early marker of osteoblast differentiation. After 5 days of cultivation, compared with the control groups, the 10^−5 ^M or 10^−6 ^M ASA VI groups showed significantly greater ALP activity (*P* < 0.05). On day 10, we observed an increase in ALP activity in all four ASA VI groups and the 10^−5 ^M ASA VI groups showed the greatest ALP activity among them, These findings indicated that 10^−5 ^M ASA VI was the optimal concentration for stimulating osteogenic differentiation in OVX rBMSCs. Thus, we adopted this concentration for use in subsequent experiments. (Fig. 3).

### Matrix mineralization

**Figure 4:**
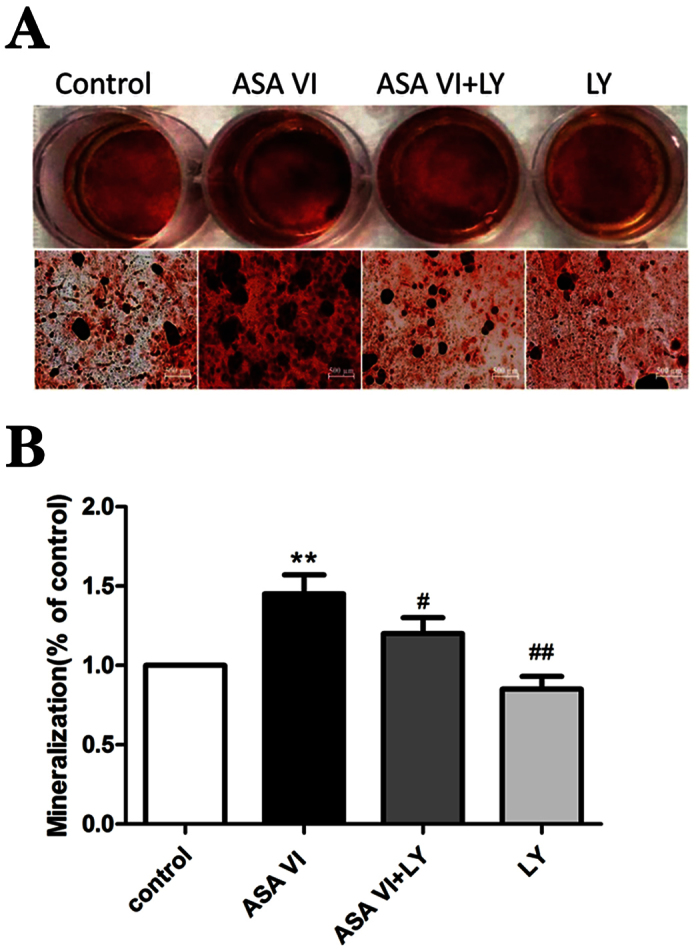
The mineralization of OVX rBMSCs. (**A**) OVX rBMSCs were seeded in 24-well plates at a cell density of 5 × 10^3^ and were treated with ASA VI (10^−5^ M) and/or LY294002 (50 μM); after 21 days, the formation of mineralized nodules was measured by alizarin red S staining. (**A**) After imaging the nodules, 10% cetylpyridinium chloride was used to dissolve the nodules, and mineralization was quantified using a Bio-Rad microplate reader. (**B**) The data are represented as the mean ± SD. **P < 0.01 vs the controls; ^#^*P* < 0.05 and ^##^*P* < 0.01 vs ASA VI 10^−5 ^M.

To assess matrix mineralization, we measured the area of calcium deposition and the absorbance index values (Fig. 4). The ASA VI treatment resulted in a significantly larger calcium deposition area compared with the controls (*P* < 0.01). In addition, cells treated with ASA VI and the PI3K inhibitor LY294002 exhibited a much smaller area of calcium deposition than did cells treated with ASA VI alone (*P* < 0.05); this value was similar to that of the control groups. These data indicated that the PI3K pathway inhibitor LY294002 interfered with the osteogenic differentiation of OVX rBMSCs induced by ASA VI.

### Real-time PCR

**Figure 5:**
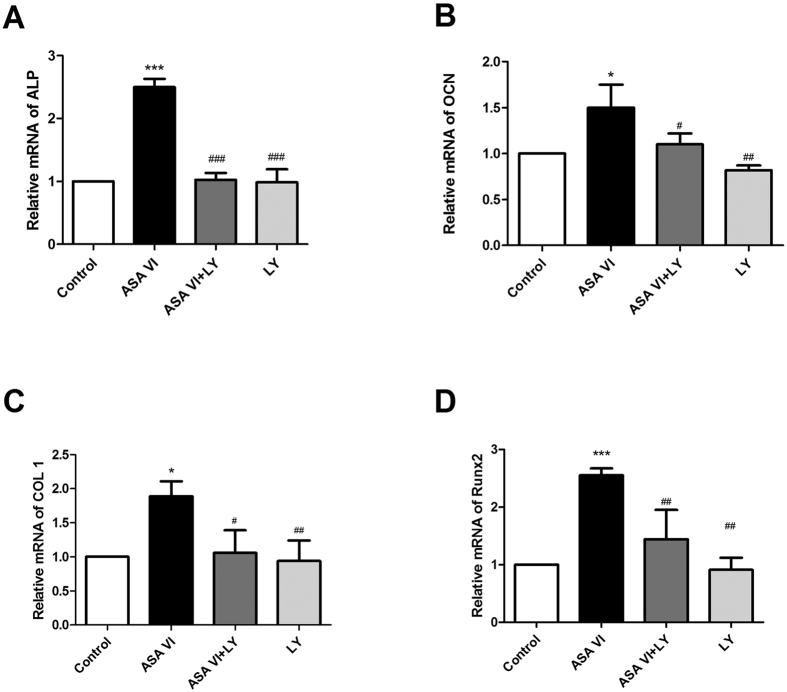
Cultured cells were treated with ASA VI 10^−5 ^M for 48 hours with or without LY294002 (50 μM). Total RNA was then extracted for quantitative PCR to assess the mRNA levels of osteogenesis-related genes, including (**A**) ALP, (**B**) OCN, (**C**) COL 1, and (**D**) RUNX2. The data are represented as the mean ± SD. *P < 0.05, **P < 0.01, and ***P < 0.001 vs the controls; ^#^*P* < 0.05, ^##^*P* < 0.01, and ^###^*P* < 0.01 vs ASA VI 10^−5 ^M.

We explored the expression of the osteogenic genes ALP, OCN, COL 1 and RUNX2 after 48 hours of culture with ASA VI and/or LY294002, as described above. Compared with the controls, the ASA VI treatment significantly enhanced the expression of osteogenic genes in OVX rBMSCs (ALP, *P* < 0.001; OCN, *P* < 0.05; COL 1, *P* < 0.05; and RUNX2, *P* < 0.001). In comparison, the treatment with both LY294002 and ASA VI yielded normal expression levels (Fig. 5).

### Western blot analysis

**Figure 6:**
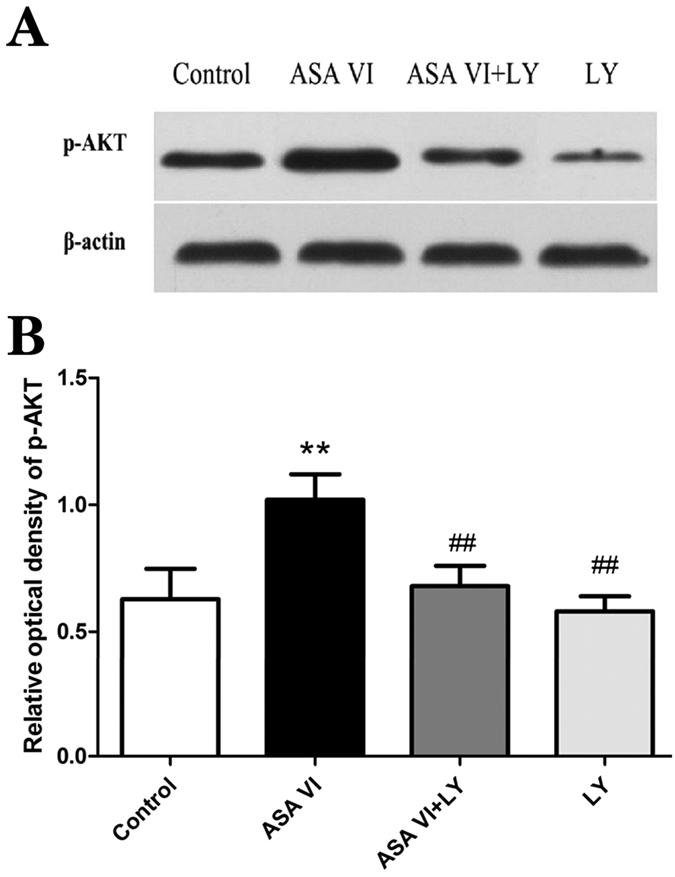
The effects of ASA VI on p-AKT expression in OVX rBMSCs. (**A**) Western blotting was used to analyze p-AKT expression after a 48-hour ASA VI 10^−5 ^M treatment, with or without LY294002. (**B**) The Western blotting results were analyzed using Quantity One Bio-Rad software. The data are represented as the mean ± SD. **P < 0.01 vs the controls; ^###^*P* < 0.001 vs ASA VI 10^−5 ^M.

The Western blot analysis indicated that ASA VI induced a significant increase in the expression of p-AKT (*P* < 0.01), while LY294002 decreased this expression (*P* < 0.01) (Fig. 6). These results demonstrate that ASA VI stimulates osteogenic differentiation in OVX rBMSCs through PI3K/AKT pathway activation.

## Discussion

ASA VI is a major active component that can be extracted from *Radix Dipsaci*, which is used in traditional Chinese medicine to enhance bone formation^19^. Previous reports have shown that *Radix Dipsaci* can alleviate OP^16^,^17^,^18^. Although *Radix Dipsaci* is a natural, stable and widely available drug, there are few reports on this compound in the literature. ASA VI has shown the ability to stimulate the proliferation and osteogenic differentiation of MC3T3-E1 cells and calvarial osteoblasts in healthy mice^20^. However, the mechanism by which ASA VI promotes ossification is unknown. Moreover, to the best of our knowledge, there are no reports on the role of ASA VI in bone formation during OP. In this study, we showed that ASA VI was involved in proliferation, osteogenic differentiation and calcification of rBMSC isolated from osteoporotic rats.

The PI3K pathway plays a major role in cell proliferation, differentiation, adhesion and apoptosis^28^, and recent research has demonstrated a close relationship between PI3K/AKT signaling and bone tissue metabolism^22^,^29^. In osteoblasts, PI3K/AKT pathway activation has been shown to stimulate proliferation and differentiation while also inhibiting apoptosis^30^,^31^. Additionally, the PI3K/AKT pathway can affect osteoclast formation^29^. There are also reports that mice deficient in AKT1 or AKT2 (two of the three mammalian AKTs)^32^,^33^ displayed serious bone defects, as well as other major developmental defects. In contrast, the activation of AKT increases bone mass by blocking osteoblastic phosphatidylinositol-3,4,5-trisphosphate 3-phosphataseexpression in mice^34^. Recent studies of PI3K signaling have underscored the involvement of the PI3K/AKT signaling pathway in OP^22^. Therefore, this pathway is critical for maintaining bone stability under both normal and pathological conditions. Accordingly, it has enormous potential as a therapeutic target for OP. Our findings show that at a concentration of 10^−5 ^M, ASA VI enhanced ALP activity in OVX rBMSCs, improved mineralization, and promoted the expression of the osteogenic genes ALP, OCN, COL 1, and RUNX2. ASA VI also enhanced the expression of p-AKT in OVX rBMSCs. These effects were inhibited by the PI3K/AKT inhibitor LY294002, indicating that the ability of ASA VI to stimulate osteogenic differentiation in OVX rBMSCs is dependent on the PI3K/AKT signaling pathway.

As a natural medicine that can promote osteogenesis, Icariin has been extensively studied, and it has been linked to the promotion of osteogenesis through different pathways, such as the BMP-2, SMAD, RUNX2 and osterix^35^, estrogen^36^, MAPK^37^ and PI3K/AKT^26^ signaling pathways. These observations indicate that a single drug can function synergistically via multiple signaling pathways, with the added benefit of some crosstalk occurring among them. Previous studies have demonstrated the ability of ASA VI to promote the expression of BMP-2 and the activation of p38 and ERK1/2, confirming that its role in promoting osteoblast differentiation is dependent on the BMP-2 pathway^20^. In this study, we showed that ASA VI promoted the osteogenic differentiation of OVX rBMSCs and that the increased expression of RUNX2 is dependent on PI3K/AKT. We believe that these two results are not contradictory. The two pathways are related to each other in terms of osteoblast differentiation. RUNX2 is one of the most important transcription factors for osteoblast differentiation and bone formation^38^. BMPs are best known for their actions as signals for bone formation^39^, and BMP-2 is capable of promoting the expression of the osteogenic gene RUNX2^40^. In turn, RUNX2 is capable of improving the activity of the PI3K/AKT signaling pathway by increasing the protein levels of PI3K p85, p110β and AKT. Furthermore, PI3K/AKT signaling enhances the DNA binding of RUNX2 and RUNX2-dependent transcription^41^. This positive feedback loop further enhances RUNX2 activity during osteoblast differentiation. Moreover, crosstalk exists between the BMP-specific SMAD and PI3K/AKT signaling pathways in the regulation of BMP-2 transcription^42^. However, although we have demonstrated that ASA VI is a natural drug that can enhance osteogenesis *in vitro*, how this compound regulates several signaling pathways remains to be elucidated.

## Conclusion

ASA VI has the ability to promote the proliferation, osteogenic differentiation and mineralization of OVX rBMSCs. Its optimal concentration is 10^−5 ^M, and its action is dependent on the PI3K/AKT signaling pathway. Therefore, ASA VI has potential as a new drug for treating women who suffer from post-menopausal OP.

## Additional Information

**How to cite this article**: Ke, K. *et al*. Asperosaponin VI promotes bone marrow stromal cell osteogenic differentiation through the PI3K/AKT signaling pathway in an osteoporosis model. *Sci. Rep.* **6**, 35233; doi: 10.1038/srep35233 (2016).

## References

[b1] Lewiecki E. M. New targets for intervention in the treatment of postmenopausal osteoporosis. Nat Rev Rheumatol. 7, 631–638 (2011). 21931340 10.1038/nrrheum.2011.130

[b2] Riggs B. L. & Melton L. J. The worldwide problem of osteoporosis: insights afforded by epidemiology. Bone. 17, 505s–511s (1995). 8573428 10.1016/8756-3282(95)00258-4

[b3] Cooper C. . Population-based study of survival after osteoporotic fractures. Am J Epidemiol. 137, 1001–1005 (1993). 8317445 10.1093/oxfordjournals.aje.a116756

[b4] Burge R. . Incidence and economic burden of osteoporosis-related fractures in the United States, 2005–2025. J Bone Miner Res. 22, 465–475 (2007). 17144789 10.1359/jbmr.061113

[b5] Harada S. & Rodan G. A. Control of osteoblast function and regulation of bone mass. Nature. 423, 349–355 (2003). 12748654 10.1038/nature01660

[b6] An Q., Wu D., Ma Y., Zhou B. & Liu Q. Suppression of Evi1 promotes the osteogenic differentiation and inhibits the adipogenic differentiation of bone marrow-derived mesenchymal stem cells *in vitro*. Int J Mol Med. 36, 1615–1622 (2015). 26497332 10.3892/ijmm.2015.2385

[b7] Chen X. D., Shi S., Xu T., Robey P. G. & Young M. F. Age-related osteoporosis in biglycan-deficient mice is related to defects in bone marrow stromal cells. J Bone Miner Res . 17, 331–340 (2002). 11811564 10.1359/jbmr.2002.17.2.331

[b8] Antebi B., Pelled G. & Gazit D. Stem cell therapy for osteoporosis. Curr Osteoporos Rep. 12, 41–47 (2014). 24407712 10.1007/s11914-013-0184-x

[b9] Nieves J. W. & Cosman F. Atypical subtrochanteric and femoral shaft fractures and possible association with bisphosphonates. Curr Osteoporos Rep. 8, 34–39 (2010). 20425089 10.1007/s11914-010-0007-2

[b10] Barrett-Connor E. . Effects of raloxifene on cardiovascular events and breast cancer in postmenopausal women. Digest of the World Core Medical Journals. 355, 125–137 (2006). 10.1056/NEJMoa062462 16837676

[b11] Anastasilakis A. D., Toulis K. A., Polyzos S. A., Anastasilakis C. D. & Makras P. Long-term treatment of osteoporosis: safety and efficacy appraisal of denosumab. Ther Clin Risk Manag. 8, 295–306 (2012). 22767993 10.2147/TCRM.S24239 PMC3387828

[b12] Reginster J. Y., Pelousse F. & Bruyere O. Safety concerns with the long-term management of osteoporosis. Expert Opin Drug Saf. 12, 507–522 (2013). 23614635 10.1517/14740338.2013.793669

[b13] Guo A. J. . Baicalin, a flavone, induces the differentiation of cultured osteoblasts: an action via the Wnt/beta-catenin signaling pathway. J Biol Chem. 286, 27882–27893 (2011). 21652696 10.1074/jbc.M111.236281 PMC3151034

[b14] Gao F., Mo X. & Li J. Analysis of genes expression profiles of icariin in treating osteoporosis of ovariectomized rats. Chinese Journal of Information on Traditional Chinese Medicine. 2, 43–45 (2013).

[b15] Li F. . Naringin prevents ovariectomy-induced osteoporosis and promotes osteoclasts apoptosis through the mitochondria-mediated apoptosis pathway. Biochem Biophys Res Commun. 452, 629–635 (2014). 25181344 10.1016/j.bbrc.2014.08.117

[b16] Niu Y. B. . Treatment of Radix Dipsaci extract prevents long bone loss induced by modeled microgravity in hindlimb unloading rats. Pharm Biol. 53, 110–116 (2015). 25243871 10.3109/13880209.2014.911920

[b17] Liu Z. G. . The osteoprotective effect of Radix Dipsaci extract in ovariectomized rats. J Ethnopharmacol. 123, 74–81 (2009). 19429343 10.1016/j.jep.2009.02.025

[b18] Niu Y. B. . Radix Dipsaci total saponins stimulate MC3T3-E1 cell differentiation via the bone morphogenetic protein-2/MAPK/Smad-dependent Runx2 pathway. Mol Med Rep. 11, 4468–4472 (2015). 25625570 10.3892/mmr.2015.3249

[b19] Li C. . Protective roles of Asperosaponin VI, a triterpene saponin isolated from Dipsacus asper Wall on acute myocardial infarction in rats. Eur J Pharmacol. 627, 235–241 (2010). 19909736 10.1016/j.ejphar.2009.11.004

[b20] Niu Y. B. . Asperosaponin VI, a saponin component from Dipsacus asper wall, induces osteoblast differentiation through bone morphogenetic protein-2/p38 and extracellular signal-regulated kinase 1/2 pathway. Phytother Res. 25, 1700–1706 (2011). 21452371 10.1002/ptr.3414

[b21] Chen J., Crawford R., Chen C. & Xiao Y. The key regulatory roles of the PI3K/Akt signaling pathway in the functionalities of mesenchymal stem cells and applications in tissue regeneration. Tissue Eng Part B Rev. 19, 516−528 (2013). 23651329 10.1089/ten.TEB.2012.0672

[b22] Xi J. C. . The PI3K/AKT cell signaling pathway is involved in regulation of osteoporosis. J Recept Signal Transduct Res. 35, 640–645 (2015). 26390889 10.3109/10799893.2015.1041647

[b23] Li C. . Asperosaponin VI protects cardiac myocytes from hypoxia-induced apoptosis via activation of the PI3K/Akt and CREB pathways. Eur J Pharmacol. 649, 100–107 (2010). 20863824 10.1016/j.ejphar.2010.08.060

[b24] Wojtowicz A. M., Templeman K. L., Hutmacher D. W., Guldberg R. E. & Garcia A. J. Runx2 overexpression in bone marrow stromal cells accelerates bone formation in critical-sized femoral defects. Tissue Eng Part A. 16, 2795–2808 (2010). 20412027 10.1089/ten.tea.2010.0025 PMC2928046

[b25] Wakitani S., Saito T. & Caplan A. I. Myogenic cells derived from rat bone marrow mesenchymal stem cells exposed to 5-azacytidine. Muscle Nerve. 18, 1417–1426 (1995). 7477065 10.1002/mus.880181212

[b26] Zhai Y. K. . Icariin stimulates the osteogenic differentiation of rat bone marrow stromal cells via activating the PI3K-AKT-eNOS-NO-cGMP-PKG. Bone. 66, 189–198 (2014). 24956021 10.1016/j.bone.2014.06.016

[b27] Zhou Y. . Effects of leukemia inhibitory factor on proliferation and odontoblastic differentiation of human dental pulp cells. J Endod. 37, 819–824 (2011). 21787496 10.1016/j.joen.2011.02.031

[b28] Erdogdu O., Nathanson D., Sjoholm A., Nystrom T. & Zhang Q. Exendin-4 stimulates proliferation of human coronary artery endothelial cells through eNOS-, PKA- and PI3K/Akt-dependent pathways and requires GLP-1 receptor. Mol Cell Endocrinol. 325, 26–35 (2010). 20452396 10.1016/j.mce.2010.04.022

[b29] Marie P. J. Signaling pathways affecting skeletal health. Curr Osteoporos Rep. 10, 190–198 (2012). 22711369 10.1007/s11914-012-0109-0

[b30] Guntur A. R. & Rosen C. J. The skeleton: a multi-functional complex organ: new insights into osteoblasts and their role in bone formation: the central role of PI3Kinase. J Endocrinol. 211, 123–130 (2011). 21673026 10.1530/JOE-11-0175 PMC3348869

[b31] Ling L. . Synergism between Wnt3a and heparin enhances osteogenesis via a phosphoinositide 3-kinase/Akt/RUNX2 pathway. J Biol Chem. 285, 26233–26244 (2010). 20547765 10.1074/jbc.M110.122069 PMC2924036

[b32] Brazil D. P., Yang Z. Z. & Hemmings B. A. Advances in protein kinase B signalling: AKTion on multiple fronts. Trends Biochem Sci. 29, 233–242 (2004). 15130559 10.1016/j.tibs.2004.03.006

[b33] Manning B. D. & Cantley L. C. AKT/PKB signaling: navigating downstream. Cell. 129, 1261–1274 (2007). 17604717 10.1016/j.cell.2007.06.009 PMC2756685

[b34] Liu X. . Lifelong accumulation of bone in mice lacking Pten in osteoblasts. Proc Natl Acad Sci USA 104, 2259–2264 (2007). 17287359 10.1073/pnas.0604153104 PMC1892939

[b35] Song L., Zhao J., Zhang X., Li H. & Zhou Y. Icariin induces osteoblast proliferation, differentiation and mineralization through estrogen receptor-mediated ERK and JNK signal activation. Eur J Pharmacol. 714, 15–22 (2013). 23764463 10.1016/j.ejphar.2013.05.039

[b36] Xiao H. H. . Flavonoids from Herba epimedii selectively activate estrogen receptor alpha (ERalpha) and stimulate ER-dependent osteoblastic functions in UMR-106 cells. J Steroid Biochem Mol Biol. 143, 141–151 (2014). 24607839 10.1016/j.jsbmb.2014.02.019

[b37] Zhou H., Wang S., Xue Y. & Shi N. Regulation of the levels of Smad1 and Smad5 in MC3T3-E1 cells by Icariine *in vitro*. Mol Med Rep. 9, 590–594 (2014). 24297369 10.3892/mmr.2013.1837

[b38] Komori T. Mechanism of transcriptional regulation by Runx2 in osteoblasts. Clin Calcium. 16, 801–807 (2006). 16679622

[b39] Rosen V. BMP2 signaling in bone development and repair. Cytokine Growth Factor Rev. 20, 475–480 (2009). 19892583 10.1016/j.cytogfr.2009.10.018

[b40] Phimphilai M., Zhao Z., Boules H., Roca H. & Franceschi R. T. BMP signaling is required for RUNX2-dependent induction of the osteoblast phenotype. J Bone Miner Res. 21, 637–646 (2006). 16598384 10.1359/JBMR.060109 PMC2435171

[b41] Fujita T. . Runx2 induces osteoblast and chondrocyte differentiation and enhances their migration by coupling with PI3K-Akt signaling. J Cell Biol. 166, 85–95 (2004). 15226309 10.1083/jcb.200401138 PMC2172136

[b42] Ghosh-Choudhury N. . Requirement of BMP-2-induced phosphatidylinositol 3-kinase and Akt serine/threonine kinase in osteoblast differentiation and Smad-dependent BMP-2 gene transcription. J Biol Chem. 277, 33361–33368 (2002). 12084724 10.1074/jbc.M205053200

